# Supplementary figures and images for: Adaptive sentiment analysis using multioutput classification: a performance comparison
Source: PeerJ Comput Sci. 2023 May 9;9:e1378. doi: 10.7717/peerj-cs.1378 (PMC10280487; doi:10.7717/peerj-cs.1378)

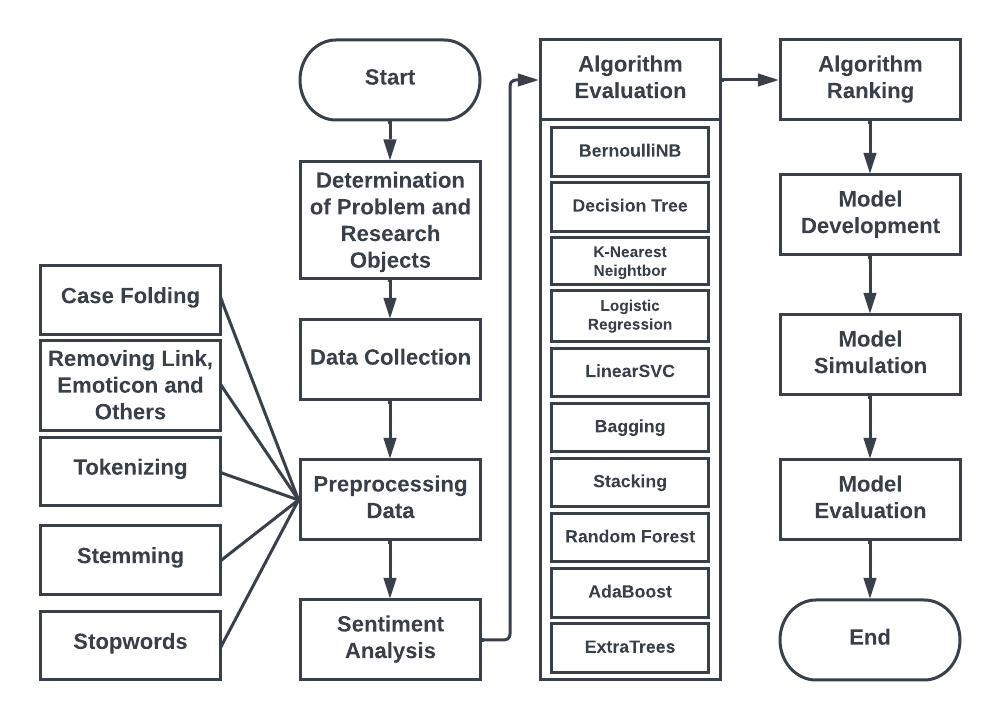

Supplement: Supplemental Information 1 — The dataset consists of 1,599 sentiment data of cryptocurrency exchange customers in Indonesia, the data consists of 2 columns namely the “review” and “sentiment” columns which are categorized into positive and negative. The function of the review column serves as data in the form of comment sentences, opinions, and suggestions that will later be learned by the model. The function of the sentiment column is to identify the category of review data between “positive” and “negative”. [file peerj-cs-09-1378-s001.zip › PeerJ/Figure 1. Research steps.png]

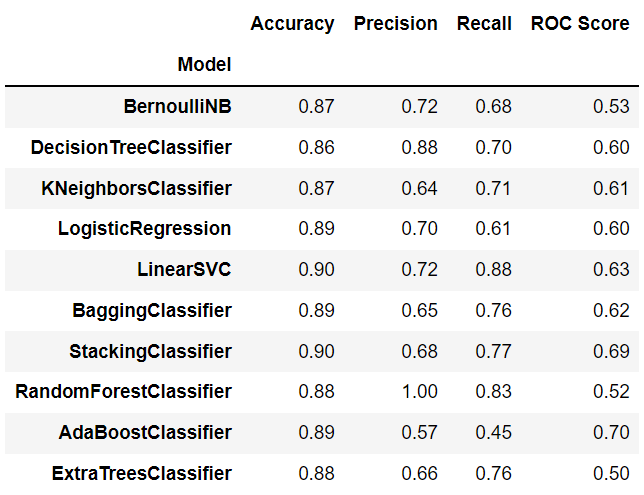

Supplement: Supplemental Information 1 — The dataset consists of 1,599 sentiment data of cryptocurrency exchange customers in Indonesia, the data consists of 2 columns namely the “review” and “sentiment” columns which are categorized into positive and negative. The function of the review column serves as data in the form of comment sentences, opinions, and suggestions that will later be learned by the model. The function of the sentiment column is to identify the category of review data between “positive” and “negative”. [file peerj-cs-09-1378-s001.zip › PeerJ/Figure 2. Algorithm rankings.png]

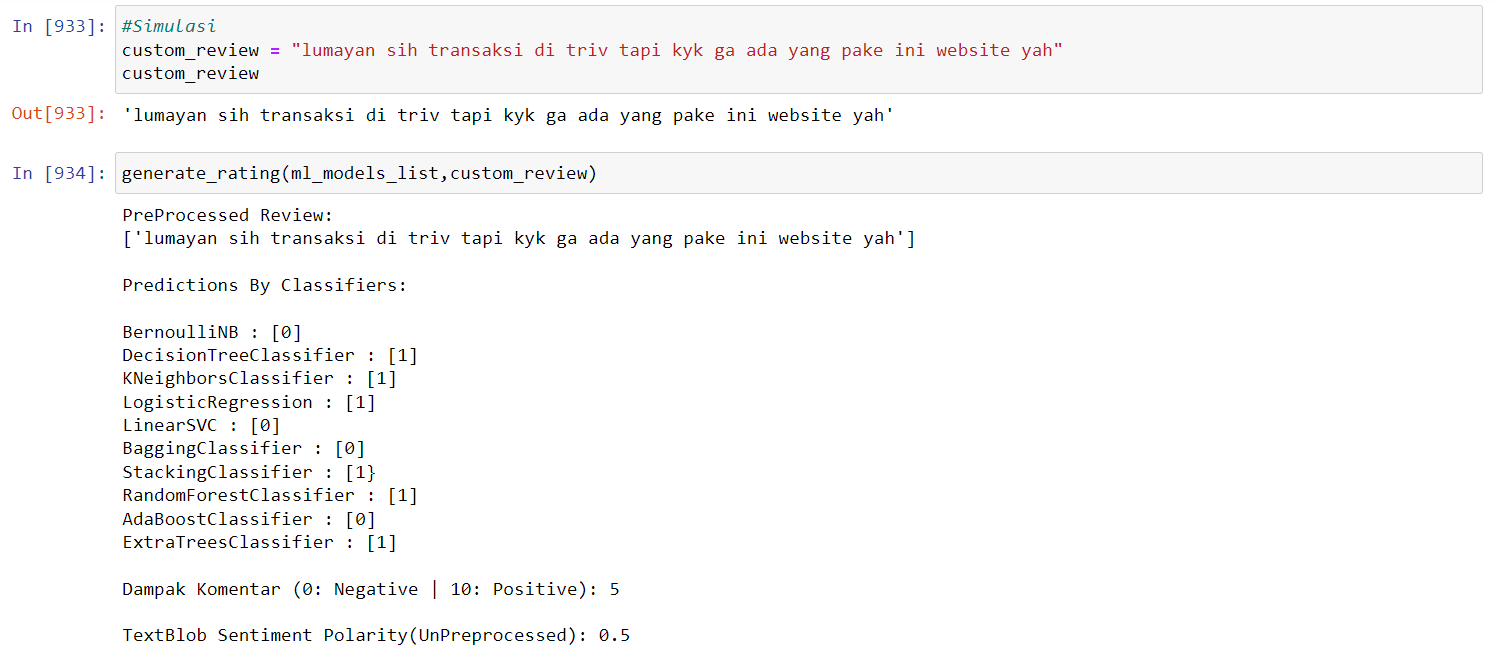

Supplement: Supplemental Information 1 — The dataset consists of 1,599 sentiment data of cryptocurrency exchange customers in Indonesia, the data consists of 2 columns namely the “review” and “sentiment” columns which are categorized into positive and negative. The function of the review column serves as data in the form of comment sentences, opinions, and suggestions that will later be learned by the model. The function of the sentiment column is to identify the category of review data between “positive” and “negative”. [file peerj-cs-09-1378-s001.zip › PeerJ/Figure 3. Model result towards neutral data (1).png]

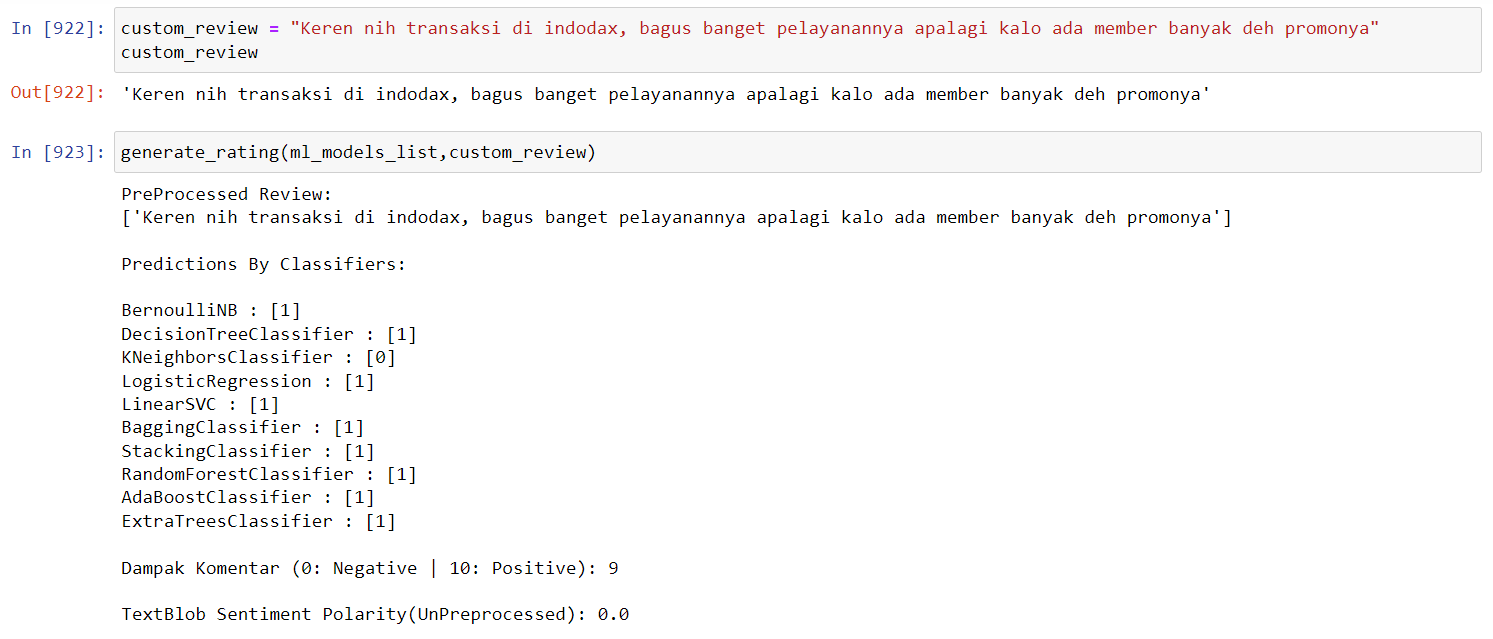

Supplement: Supplemental Information 1 — The dataset consists of 1,599 sentiment data of cryptocurrency exchange customers in Indonesia, the data consists of 2 columns namely the “review” and “sentiment” columns which are categorized into positive and negative. The function of the review column serves as data in the form of comment sentences, opinions, and suggestions that will later be learned by the model. The function of the sentiment column is to identify the category of review data between “positive” and “negative”. [file peerj-cs-09-1378-s001.zip › PeerJ/Figure 4. Model result towards positive data (1).png]

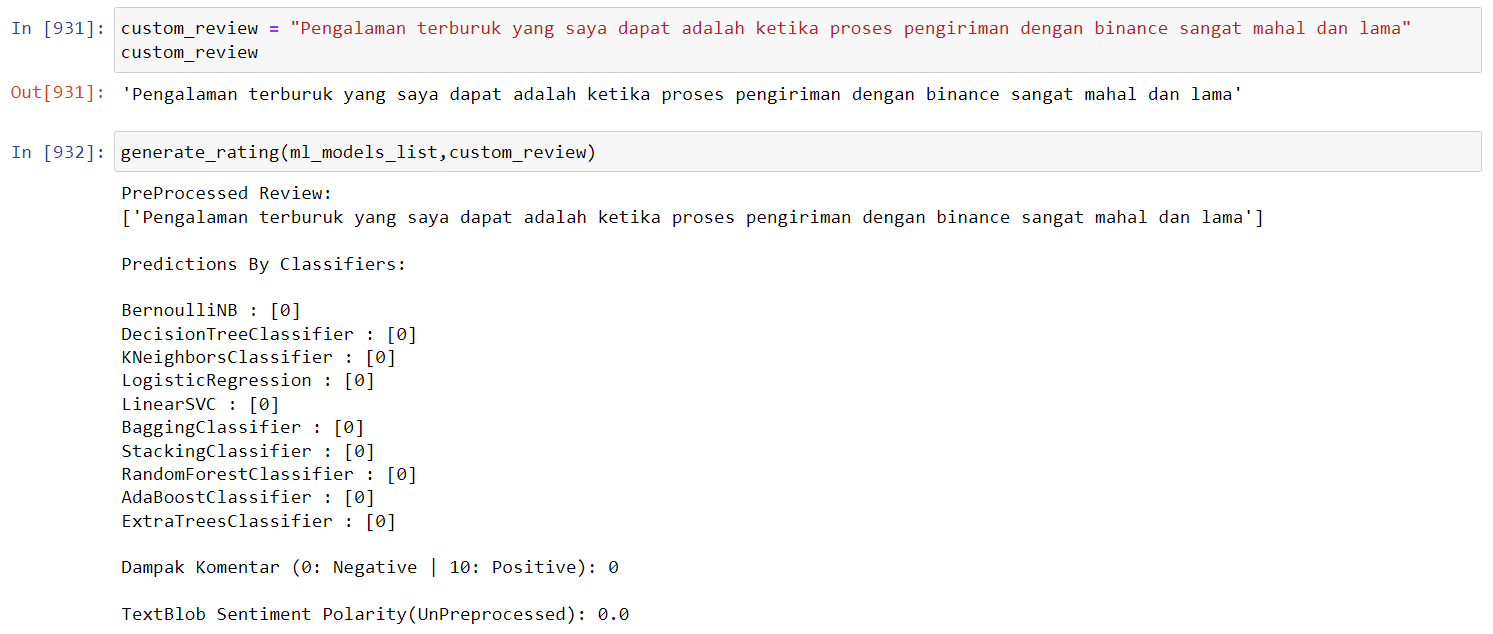

Supplement: Supplemental Information 1 — The dataset consists of 1,599 sentiment data of cryptocurrency exchange customers in Indonesia, the data consists of 2 columns namely the “review” and “sentiment” columns which are categorized into positive and negative. The function of the review column serves as data in the form of comment sentences, opinions, and suggestions that will later be learned by the model. The function of the sentiment column is to identify the category of review data between “positive” and “negative”. [file peerj-cs-09-1378-s001.zip › PeerJ/Figure 5. Model result towards negative data (1).png]
